# Early bioprosthetic valve calcification with alfacalcidol supplementation

**DOI:** 10.1186/1749-8090-8-11

**Published:** 2013-01-16

**Authors:** Tamaki Takano, Takamitsu Terasaki, Yuko Wada, Noburou Ohashi, Kazunori Komatsu, Daisuke Fukui, Jun Amano

**Affiliations:** 1 Department of Cardiovascular Surgery, Shinshu University School of Medicine, 3-1-1 Asahi, Matsumoto, 390-8621, Japan

**Keywords:** Prosthesis, Biomaterials, Aortic valve, Replacement, Calcification, Heart valve, Bioprosthesis

## Abstract

We report a case of early bioprosthetic valve calcification in a 76 year-old woman who had received supplementation with alfacalcidol, an analogue of vitamin D, for 3 years after her initial valve replacement. She underwent aortic valve replacement at the age of 71 and subsequently complained of shortness of breath. Ultrasonic cardiography revealed severe aortic stenosis and we performed a second aortic valve replacement with a bioprosthesis. Histopathologic and x-ray examination showed calcification on the explanted valve. She had not presented with any known risk for early bioprosthetic calcification, suggesting that vitamin D supplementation may accelerate calcification of bioprosthetic valves.

## Background

Durability of bioprosthetic valve is increasing because valve tissue treatment and valve structure is improving from day to day, and actuarial freedom from explants due to valve deterioration at 20 years is reported as 81.5% in patients older than 64 years [1]. However, there are some papers of early bioprosthetic valve deterioration in the older patients, and risk factors for early deterioration of bioprosthesis were not completely uncovered yet [2,3]. It is important to report possible risk to understand mechanism of early valve deterioration. We here describe a case of early bioprosthetic valve calcification with alfacalcidol supplementation.

## Case

A 76 year-old women visited a hospital complaining of shortness of breath on effort. She had undergone aortic valve replacement (AVR) for aortic stenosis with a 21 mm Carpentier-Edwards Perimount (CEP, model 2900, Edwards Lifesciences, CA, USA) concomitant with coronary artery bypass grafting at the age of 71, 5 years previously. Echocardiography (UCG) showed aortic stenosis with a maximum pressure gradient of 66 mmHg and an aortic valve area (AVA) of 0.80 cm^2^. Shortness of breath worsened and orthopnea appeared 2 months after her initial symptoms started and 1 month after the initial UCG. She was admitted to the hospital and given intravenous dobutamine for congestive heart failure. UCG showed rapid progression of aortic stenosis with a maximum pressure gradient of 110 mmHg, an AVA of 0.30 cm^2^, and a fractional shortening (FS) of 37%. She was transferred to our hospital for surgery. She had been taking 1 μg/day of alfacalcidol for 3 years for osteoporosis, starting 2 years after the initial AVR, and amlodipine and levothyroxine for hypertension and hypothyroidism, respectively. She also had a history of right breast cancer. Blood chemistry showed a corrected serum calcium (serum Ca-Albumin + 4) concentration of 9.1 ± 0.7 mg/dl (with a range of 8.0 to 10.2 mg/dl) at the outpatient clinic before hospital admission.

**Figure 1:**
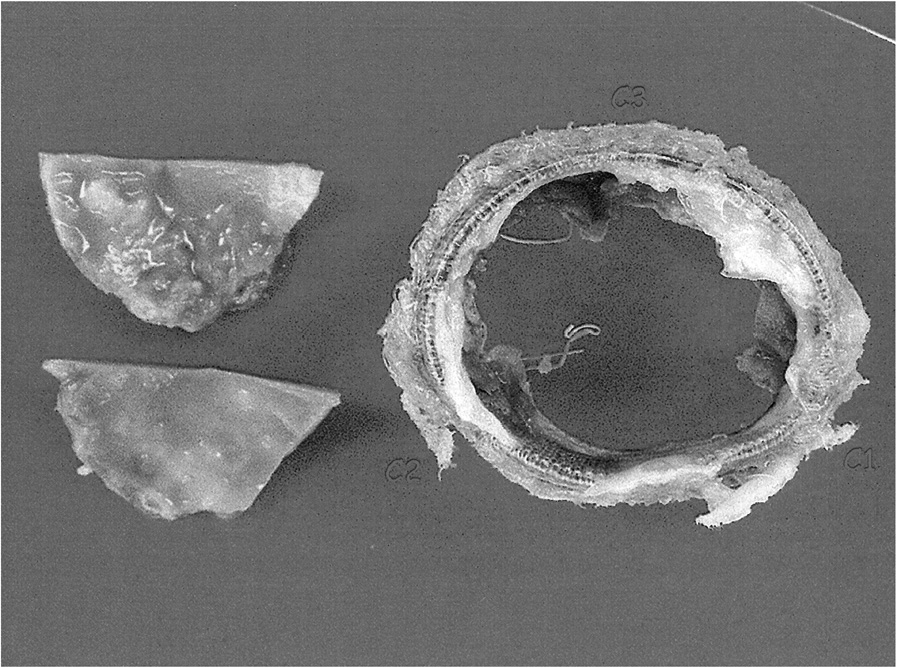
**Explanted Carpentier-Edwards Perimount valve.** Thickening and sclerosis are observed in the incised cusps.

We performed emergency surgery. After cardiopulmonary bypass (CPB) was initiated with ascending aorta and right atrial cannulation, the ascending aorta was cross-clamped and aortotomy was done at 2 cm distal to the saphenous vein graft anastomosis. All leaflets of the CEP were sclerotic [Figure 1] and the left and non-coronary cusps were fused. We excised the leaflets to ensure full inspection of the aortic annulus because the stents of the valve strongly adhered to the aortic wall and then removed the remnant rigid structures of the CEP. No abscess or necrosis was found around the prosthesis. A new 19 mm Epic Supra bioprosthesis (St. Jude Medical, Inc., MN, USA) was implanted in a supra-annular fashion using 14 mattress sutures with spaghetti after the intimal defect at the annulus of the left coronary cusp was repaired with autologous pericardium. The patient was weaned from CPB without difficulty under continuous administration of low dose cathecholamine. The post-operative course was uneventful. UCG showed a maximum pressure gradient of 27 mm Hg and a slightly reduced FS of 26% at 10 days after the re-operation but the FS had recovered to 35% by 16 days after the re-operation.

**Figure 2:**
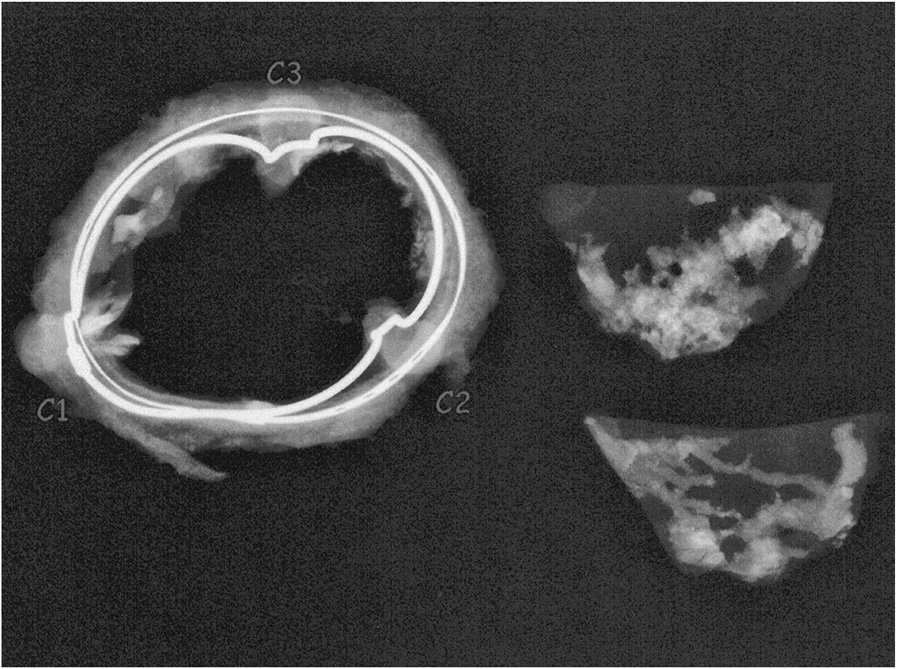
Acinar and linear calcifications are found in the cusps by X-ray examination.

Histopathologic examination demonstrated moderate to severe calcification and hyalinization on the explanted valves. X-ray examination showed acinar calcification in the cusps of the explanted valves [Figure 2]. There was infiltration of neutrophils, histiocytes and lymphocytes. Arterial blood gas analysis showed a plasma free Ca^2+^ of 4.0-4.4 mg/dl, which was just below the normal value of 4.8-5.4 mg/dl before and after surgery.

## Discussion

This report describes a case of early cuspal calcifications of a bioprosthetic valve in an elderly woman who had been taking alfacalcidol for 3 years for osteoporosis after the initial valve replacement. We could not find any reports of early prosthetic valve calcification in patients taking vitamin D supplementation, whereas it has been reported that administration of vitamin D caused the development of aortic stenosis in rabbits [4]. Calcification in bioprosthetic valves is accelerated by younger recipient age, glutaraldehyde fixation, high mechanical stress and chronic kidney disease including dialysis [5,6]. In a recent paper evaluating 25 years of experience with the CEP (Model 6900) in the mitral position, structural valve deterioration occurred more frequently in recipients under 60 years of age compared to those over 60 and was unrelated to the etiology of the native valve disease [7]. The patient in this case report had received initial valve replacement at the age of 71 and did not have chronic kidney disease or other risks for early valve calcification. This case suggests that vitamin D may accelerate calcification of bioprosthetic valves. Vitamin D is sometimes used for treatment of osteoporosis, and perhaps should be used carefully after bioprosthetic valve replacement because the age of patients at valve replacement has increased recently, so that more patients who receive bioprosthetic valves also require treatment for osteoporosis due to increased age.

The mechanism of calcification on bioprosthetic valves is not completely understood. Calcification occurs within nonviable connective tissue cells which are treated by glutaraldehyde through a reaction of calcium-containing extracellular fluid with membrane-associated phosphorus. Glutaraldehyde-treated cells are unable to pump out calcium from the cytoplasm and inter-cellular structures, which are high in phosphorus and can bind calcium, whereas the membranes of normal healthy cells pump calcium out to maintain a level 1000 to 10000 times lower than plasma or extracellular calcium concentrations [2]. Higher extracellular calcium concentrations may accelerate bioprosthetic valve calcification. Serum calcium concentration in the present case was 9.1 ± 0.7 mg/dl, within the normal range of 8.0 to 10.2 mg/dl, although the patient had been taking 1 μg/day of alfacalcidol for 3 years. Plasma free calcium ion was 4.0-4.4 mg/dl in arterial blood gas analysis before and after the re-AVR. High calcium concentration did not seem to be directly related to early calcification in the present case. Liao K et al. reported that the administration of diphenylhydantoin, a vitamin D antagonist, for 45 days inhibited calcification of bovine pericardium implants treated with glutaraldehyde in rats. Vitamin D is considered to potentially stimulate osteocalcin synthesis and it also activates calcium-binding proteins and increases calcium absorption [8]. Vitamin D might have directly accelerated prosthetic valvular calcification through osteocalcin without increasing serum calcium concentration in the present case. It was also recently reported that osteoporosis treatment with bisphosphonates or calcitonin or selective estrogen receptor modulators inhibited progression of aortic stenosis [9]. These authors speculate that the inhibition was due to changes in vitamin D and parathyroid hormone levels and that osteopontin mediated the effect of bisphosphonates on calcification of the aortic valve. Further studies would be warranted to understand the effects of regulators in bone metabolism, which include vitamin D and its analogues, on calcification of bioprosthetic valves.

## Conclusion

We report early bioprosthetic valve calcification in a patient taking alfacalcidol supplementation, although serum calcium concentrations in the patient had been within normal limits. This case suggests the possibility that vitamin D may accelerate calcification of bioprosthetic valves.

## Consent

Written informed consent was obtained from the patients for publication of this Case report. Copies of the written consent forms are available for review by the Editor-in-Chief of this journal.

## Abbreviations

AVR: Aortic valve replacement; CEP: Carpentier-Edwards Perimount; UCG: Echocardiography; AVA: Aortic valve area; FS: Fractional shortening; CPB: Cardiopulmonary bypass.

## Competing interests

All the authors have read the manuscript and have approved of its submission. The authors report no competing interest.

## Authors’ contributions

TT presented design of the case report and completed the manuscript. TT, YW, ON, KK and DF are in charge of patient care. JA directed all the work. All authors read and approved the final manuscript.
